# Re-evaluating the relationship between youth with HIV and BMI in an age of increasing rates of overweight and obese youth

**DOI:** 10.1186/s13104-024-06741-8

**Published:** 2024-04-01

**Authors:** Samantha V. Hill, Jiaying Hao, Mia Newlin-Bradner, Dustin M. Long, Henna Budhwani, Tina Simpson

**Affiliations:** 1 University of Alabama at Birmingham, Birmingham, AL USA; 2 Florida State University, Tallahassee, FL USA; 3 Tulane University, New Orleans, Louisiana USA

**Keywords:** Youth with HIV, BMI, Metabolic disease, Integrase inhibitors

## Abstract

**Background:**

Newer antiretrivirals (ART) have shifted the metabolic experiences of people with HIV (PWH) from those of wasting syndrome to increases in body mass index (BMI). This study sought to examine the relationship between BMI and ART use among youth with HIV (YWH).

**Methods:**

Charts from YWH ages 10–24 with at least two documented BMIs at least 6 months apart between 2017 and 2020 were included (N = 44). Statistical analyses were conducted in SAS 9.4.

**Results:**

Clients were predominately African American (66%) males (73%) aged 19–24 years (64%), with men having sex with men (48%) being the most common mode of transmission. YWH on non-integrase inhibitor (INSTI) regimens had greater absolute increases in BMI compared to those on INSTI regimens (p = 0.03). Fourteen percent of clients using INSTI experienced an increase in BMI class from normal to overweight or overweight to obese; no non-INSTI users changed BMI class. Time since diagnosis and BMI change due to weight gain were positively associated (p = 0.03) among behaviorally-acquired YWH.

**Conclusions:**

Increasing BMI and changing BMI classes may be more likely among YWH using INSTI. More longitudinal studies inclusive of diet and exercise profiles are needed to understand the relationship between INSTI and YWH BMI.

## Introduction

Forty years into the HIV epidemic has brought innovations in antiretroviral treatments (ART) that have transformed a once deadly infection into a lifelong chronic disease, with cardiovascular disease becoming the leading cause of death among PWH [1, 2]. New classes of ART (e.g. integrase inhibitors (INSTI)), have made previously complicated regimens into manageable regimens with daily pills (e.g. bictegravir/emtricitabine/tenofovir alafenamide) or long acting injectables (e.g. cabotegravir/rilpivirine) [3].

Estimates reveal between 50% and 66% of PWH are overweight and one fourth of PWH are obese [2, 4]. Lifestyle factors in combination with ART such as non-nucleoside reverse transcriptase inhibitors (NRTIs) and protease inhibitors have long been shown to impact body mass index (BMI) among PWH. Even newer INSTI have been associated with truncal fat development and weight gain (both of which may be more prominent in certain races, ethnicities, and genders) [5]. Coupled with use of ART which can cause lipodystrophy, dyslipidemia, and insulin resistance; PWH who are overweight or obese have a greater risk of developing metabolic syndrome compared to people without HIV, possibly due to increases in inflammation caused by the intersection of being overweight/obese and having HIV [2, 4]. Studies show that 18% of PWH who are normal weight at ART initiation become overweight within 3 years [2] and suggest that the prevalence of metabolic syndrome is not dependent on one etiology, but is associated with how each individual responds to HIV and their treatment [6]. Furthermore, HIV-related metabolic changes accumulate and lead to an increased risk of cardiovascular diseases later in adulthood [7].

The greatest incidence of HIV occurs in individuals 30 years and younger [8], with adolescents and young adults (AYA) 13–24 years old accounting for 65% of PWH. With increasing numbers of new HIV diagnoses among younger individuals who are able to live a long and full life secondary to advances in ART, understanding the relationship between ART use and body mass index (BMI) becomes more crucial than ever.

Prior BMI studies have mostly focused on PWH aged 18 and older and found factors such as race (African American), gender (female), lower socioeconomic status, and length of time on ART as predictors of being overweight and obese [2, 4]. One study focused on behaviorally-acquired young women found that NNRTIs and protease inhibitors (PIs) were associated with increased metabolic factors (obesity, dyslipidemia, glucose abnormalities) [9]. Another study focused on perinatally-infected youth aged 7–24 found that while these youth had lower BMIs compared to seronegative controls, they also had higher rates of dyslipidemia and altered glucose metabolism [10]. However, no studies have explored the relationship between obesity with newer ART released in the past 10 years. Thus, the purpose of this cross-sectional exploratory study is to examine the relationship between ART use, specifically INSTI, and obesity in YWH aged 10–24.

## Methods

### Setting

Data were collected from a Ryan White Part D and B funded HIV clinic located within an academic center in Alabama. The clinic primarily serves mothers, perinatally-exposed infants, pediatric populations with HIV from birth to 24, and young adults up to age 30 who reside in Alabama.

### Data

One hundred and thirty-nine clients, ages between 10 and 24 years, contributing 933 clinical visits between 2017 and 2020 were collected from one clinic’s electronic medical records, containing basic information including demographics, health status, laboratory, sexually transmitted infection (STI) coinfections, ART status, and service date. Charts meeting the following criteria were included: (1) YWH ages 10–24 years, (2) received HIV care at Alabama Ryan White Part D clinic between 2017 and 2020, and (3) had at least two documented visits at least 6 months from each other. Where multiple visits fit this classification, the first two documented visits at least 6 months apart were used. Client charts where the time between baseline visit and the next data point to include BMI was greater than 1 year were excluded.

### Variables

Independent variables (demographic information and antiretroviral type) were collected from charts and included the following variables: age (years) (categorized into 10–14, 15–18 and 19–24), race, sex, and mode of transmission. Antiretroviral use per client at each included visit was obtained from chart review. Dependent variables included systolic blood pressure (BPS), diastolic blood pressure (BPD), overweight and obese body mass index (BMI), viral load (VL), and CD4 count.

### Primary outcome

#### BMI

For initial visits where there was not a height or weight recorded, the height and weight from the next visit that was within 3 months of the initial visit was included. BMI was categorized into 3 groups: normal, overweight, and obese. For clients 19 years and older, BMI < 25 was defined as normal, BMI between 25 and 29.9 was defined as overweight, any BMI equal to or above 30 was defined as obese. For clients 18 years and under, Centers for Control and Prevention (CDC) BMI-for-age growth charts were referred to for BMI distributions [11]. Normal weight was defined as BMI between the 5th and 85th percentiles. Overweight was defined as BMI located between the 85th to 95th percentiles. Obese was defined as BMI at or above the 95th percentile.

### Secondary outcomes

#### Hypertension

Normal blood pressure was defined as BPS and BPD under 120 and under 80. Pre-hypertension was defined as BPS between 120 and 129 or BPD between 80 and 85. Hypertension was defined as BPS of 130 or greater or BPD of 85 or greater.

### Statistical analysis

Descriptive statistics are provided in Table 1 (demographics) and Table 2 (health values). For estimating the association between BMI change and INSTI use, ANOVA test was used. BMI change was measured by new visit BMI minus baseline visit BMI. Linear regression models were used to examine associations between BMI change in both perintally and behaviorally acquired YWH. Change in BMI class (e.g. from normal BMI to overweight BMI) was also examined and stratified by age (15–18 years vs. 19–24 years) and HIV risk factor (perinatal vs. behaviorally acquired). Fisher’s exact test was performed due to the small sample size. Contingency tables and linear regressions examined associations between length of time on INSTI since diagnosis and change in BMI class among those behaviorally diagnosed. For the association between INSTI use and other health variables, chi-square test was used. All analyses were conducted with SAS 9.4.

**Table 1:** Demographics of Adolescents 15-24yo with HIV

	Frequency *N* = 44	Percent
Age	19.73±2.7	
15 to 18 years	16	36.36
19 to 24 years	28	63.64
Sex assigned at birth		
Female	12	27.27
Male	32	72.73
Race		
AA	29	65.91
White	11	25
Other	4	9.09
HIV risk factor*		
MSM	21	47.73
Perinatal	18	40.91
Other	4	9.09
Unknown		

*1 person was MSM and IDU

**Table 2:** Baseline HIV and Metabolic Data for Adolescents 15-24yo with HIV

	Baseline	6 months
	Frequency (percent) N = 44 (100)	Frequency (percent) N = 44 (100)
VL level		
≤ 20	19 (47.5)	23 (59.0)
21–50	1 (2.5)	4 (10.3)
51–200	5 (12.5)	0 (0)
> 200	16 (41.46)	12 (30.8)
CD4 + cell count		
≤ 250	11 (27.5)	6 (15.0)
> 250	29 (72.5)	34 (85.0)
BMI class		
Normal	25 (56.8)	24 (54.5)
Overweight	8 (18.2)	8 (18.2)
Obese	7(15.9)	8 (18.2)
Hypertension		
No	14 (48.3)	
Pre-hypertension	13 (44.8)	
Yes	2 (6.9)	

## Results

### Demographics

Forty-four clients accounting for 323 clinical visits were included. Clients were predominately African American (66%) males (73%) aged 19–24 years (64%), with the most common mode of HIV acquisition being men who have sex with men (48%) (Table 1). No clients were 10–14 years old.

Table 2 displays baseline viral load, CD4 count, and metabolic data. At baseline, 8 (18%) patients were overweight and 7 (16%) were obese. Twenty-seven (66%) clients were on INSTI at baseline while 31 (70%) clients were on INSTI at 6 months with bictegravir/emtricitabine/tenofovir alafenamide and elvitegravir/cobicistat/emtricitabine/tenofovir being the most frequently used ARTs.

### Regression results

**Fig. 1:**
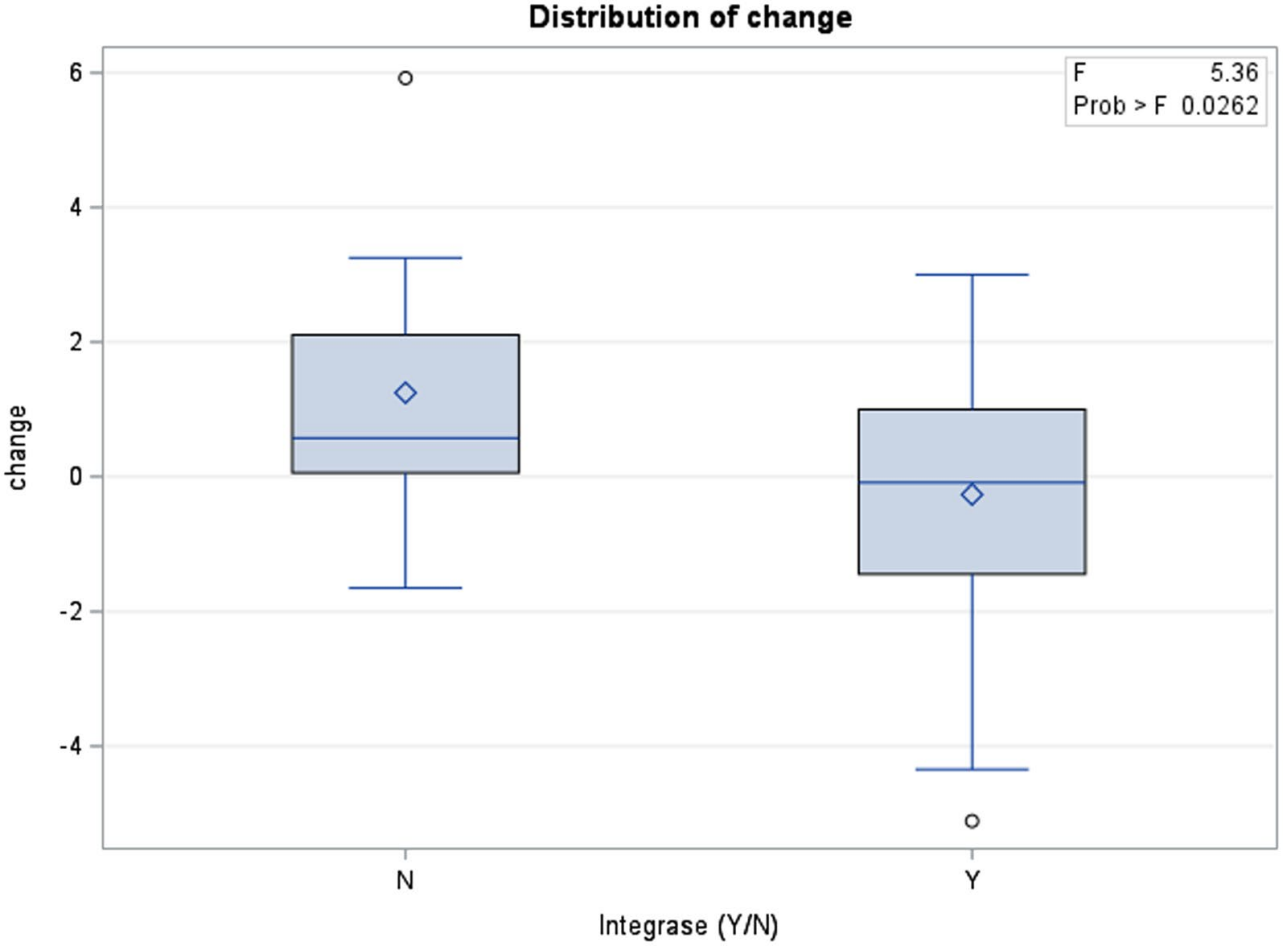
Absolute Change in BMI between Integrase Use Groups. This figure compares the change in BMIs over a 6 month period between clients using integrase inhibitors and non-integrase inhibitors. Use of non-integrase inhibitors was associated with an increase in BMI by 1.5. Use of integrase inhibitors was associated with a change in BMI decreasing by 0.2

Four patients did not have two BMIs at least 6 months apart, resulting in the sample size for regression models being N = 40. Non-INSTI users had higher change in BMI (difference = 1.5 in BMI, p = 0.03) compared with the INSTI users (Fig. 1). There were no differences between changes in BMI when comparing 15–18 year olds to 19–24 year olds (p = 0.73) after adjusting for INSTI usage. There was no association between BMI change and time between each participant’s visit (p = 0.58). Among behaviorally acquired YWH, there was a positive association between the time since diagnosis and BMI change associated with weight gain (p = 0.03).

### Change in BMI class and other variables

Changes in BMI class were observed with INSTI use, with four (15%) of the INSTI users moving from normal BMI to overweight BMI (N = 2, 5%), or from overweight BMI to obese BMI (N = 2, 5%) (Table 3); however this difference was not statistically significant (p = 0.3). There were no non-INSTI users who upgraded BMI class. There was no significant difference in change in BMI class when stratified by age group (p = 0.46 for 15–18 year olds and p = 1.0 for 19–24 year olds) or BMI class based on INSTI use when stratified by perinatally-acquired (p = 1) vs behaviorally-acquired HIV (p = 0.2).

**Table 3:** Absolute Change in BMI and BMI Class with Integrase Inhibitor Use over 6-month Period

Integrase use	Number (%) of patients whose BMI increased over 6 months	Number (%) of patients whose BMI changed classes*	P-value
No	13 (100%)	0 (0%)	0.28
Yes	23 (85.19%)	4 (14.81%)	

^*^BMI classes changed from either normal weight to overweight or from overweight to obese

There was no statistical relationship between blood pressure and INSTI use (p = 0.4). Nor was there a statistical relationship between CD4 count or viral load and BMI.

## Discussion

In this exploratory study, we sought to examine the relationship between ART use and BMI among a small sample of YWH ages 15–24 years old engaged in HIV care. A comparison of YWH on INSTI compared to those not on INSTI revealed a significant increase in BMI without a change in BMI class from baseline to 6 months in the non-INSTI group. However, YWH in the INSTI group were more likely to change BMI class (normal to overweight and overweight to obese) at 6-month follow up and there was a positive relationship between length of time on INSTI and weight gain with YWH. Thus, these findings support prior research demonstrating greater weight gain among PWH utilizing an INSTI based regimen in both treatment naïve individuals [12–16] and individuals who have switched from a PI or NNRTI based regimen to an INSTI regimen [14, 15, 17, 18].

Furthermore, these findings are also similar to other studies which reveal the multi-faceted nature of the association between INSTI use and BMI. Two studies comparing different classes of ARTs to INSTI (NNRTI to raltegravir and atazanavir/ritonavir and darunavir to raltegravir) over 156 weeks and 96 weeks respectively revealed similar increases in BMI among all medication classes [19, 20]. And while one study revealed raltegravir had increased fat gain compared to NNRTI, another found both classes had similar amounts of fat gain that contributed to increased BMI [19, 20]. Another study of adult PWH compared INSTI (raltegravir and dolutegravir), protease inhibitor (darunavir) and NNRTI (rilpivirine) and found all ART, except rilpivirine, had a significant increase in BMI at 1 year and adjustment for multiple confounders (age, sex, CD4 count, CDC stage, HIV viral load, presence of lipodystrophy/lipoatrophy, ART duration < or ≥ 3 years, and initial BMI class) diminished differences between the ART [21].

Thus, while these studies demonstrated a relationship between weight gain and INSTI use, potential confounders including age, initial BMI, CD4 count, HIV viral load, gender and ethnicity can influence this relationship [12, 13, 16, 21–24]. For instance, several studies investigating the effects of ART in PWH have observed a heightened risk of weight gain after the initiation of ART in women PWH [15, 23–27]. The Women’s Interagency HIV Study (WIHS) evaluated the impact of women switching to or adding INSTI and found that those who added or switched to INSTI had a greater increase in body weight and BMI over an 18-month period compared to women who remained on non-INSTI regimens [16]. While there were no significant differences between INSTI and non-INSTI in the current study regarding gender distribution, the overall percentage of women in this sample was low. The lack of female participants in this sample therefore may have masked potential BMI class changes within the INSTI group.

The age range represented in this study is one in which there is a paucity of literature investigating the effects of ART. Weight and height growth impairment are well-documented manifestations of HIV infection in perinatally-acquired YWH with many adolescents experiencing delayed pubertal development [29, 30]. While ART has been demonstrated to improve the anthropometric profile, many perinatally-acquired adolescents still experience height impairment in comparison to youth without HIV [3, 29–31]. Factors such as age at diagnosis; age at initiation of ART use; duration of infection; duration of ART therapy use; differences in ART regimens; and immunologic status may all influence YWH growth and development. Using data from the Collaborative Initiative for Paediatric HIV Education and Research (CIPHER) global project demonstrated that perinatally-acquired YWH with later ART initiation (i.e. after age 5) maintained average heights for age that were lower than early-ART treated YWH [7]. As was mentioned above, the age at diagnosis and duration of ART therapy were not documented in the current study. Additionally, this sample included both perinatally- and behaviorally-acquired YWH. Thus, it is possible residual impairments in growth as well as variation in the factors amongst the participants affecting growth response may be a confounder.

## Limitations

Although adding an updated picture of new INSTI use in YWH, this study’s primary limitation is its small sample size. As observed in the previously mentioned studies, there are multiple factors that can influence BMI associated with ART use, and several of those were captured. This sample consisted of African American males with low viral loads and normal CD4 counts. As such, the frequently documented changes in weight and BMI associated with INSTI may not have been as pronounced for this group. Additionally, some of the heterogeneity in the literature investigating weight gain associated with INSTI is due to some evidence of differences in weight gain related to specific INSTI [22, 24, 33]. The age range of the individuals included in this study may have introduced greater variability into the response to INSTI vs. non-INSTI regimens. The impact of HIV on growth pattern and onset of puberty as well as the growth response to ART therapy are highly variable depending on many factors that were not documented in the current study. Furthermore, changes in weight can be assessed by a variety of methods and thus reliance on a single method may not offer a comprehensive understanding. Lastly, this was a cross-sectional study that was not able to capture the multifactorial nature of each individual’s life. Future studies should be designed to capture activities of daily living including nutrition, exercise, and stressors such as work, school, and home life.

## Conclusion

Developments in ART resulting in improved immunologic recovery as well as earlier detection and treatment have dramatically improved morbidity and mortality in PWH [9, 28]. These improvements in ART therapy in addition to the continued increase in American BMI have resulted in the increased prevalence of overweight and obesity among PWH [16, 22, 25]. Evidence has shown that PWH on ART are at increased risk of developing cardiovascular disease and metabolic syndrome [7, 31–33] and the presence of excess body weight puts these individuals at even greater risk; however literature is lacking pertaining to both the effects of newer INSTI on BMI and the relationship between YWH on INSTI to BMI [1, 2, 9]. As ART continues to evolve, more investigation into the complexities of body composition changes associated with ART therapy, especially in YWH, must be pursued to prevent further increases in metabolic-disease related morbidity.

## Data Availability

The dataset supporting the conclusions of this article is available upon request to Samantha Hill, Samantha.hill.med@gmail.com.
